# Possible Genetic Links Between Solitary Fibrous Tumor and Pancreatic Cancer: A Rare Case of Solitary Fibrous Tumor and Pancreatic Cancer Concurrence

**DOI:** 10.7759/cureus.68529

**Published:** 2024-09-03

**Authors:** Ayman Naser, Ala'a Mohammad, Siham Younes, Ala'a Qashou, Zaina Abduljalil, Hanan Al-Asbhi

**Affiliations:** 1 Surgical Oncology, Al Bashir Hospital, Amman, JOR; 2 Faculty of Medicine, Jordan University Hospital, Amman, JOR; 3 Surgery, Al Bashir Hospital, Amman, JOR

**Keywords:** tp53, pancreatic cancer, solitary fibrous tumor, immunohistochemistry, genetics

## Abstract

Pancreatic ductal adenocarcinoma is the most prevalent form of pancreatic cancer, originating in the duct lining of the pancreas. The simultaneous occurrence with a solitary fibrous tumor (SFT) represents an unexpected finding. We present a case involving a 64-year-old female with synchronous pancreatic cancer and SFT. The patient initially experienced severe abdominal pain, visible jaundice, and itching. Diagnostic imaging revealed a mass in the head of the pancreas and a soft tissue mass in the right hemipelvis. Further investigations included histological examination, immunohistochemistry, and genetic testing. Subsequently, the patient underwent appropriate management, which involved the excision of both masses and radiochemotherapy. The discussion focuses on the genetic linkages in this rare presentation, aiming to identify treatment connections for both tumors. Throughout this case report, our aim is to contribute to enriching the limited literature with new insights and underscore the importance of identifying genetic linkages between both tumors which may lead to more effective management strategies and better treatment outcomes.

## Introduction

Pancreatic ductal adenocarcinoma (PDAC) accounts for over 90% of all pancreatic malignancies, originating from the epithelial cells lining the pancreatic duct, displaying gland-like features [1]. According to the Jordanian cancer registry, pancreatic cancer accounts for 1.4% of cancers in Jordan [2]. Pancreatic cancer may be associated with other tumors such as breast, melanoma, and other gastrointestinal malignancies [3]. In this case, we explore the simultaneous occurrence of a benign solitary fibrous tumor (SFT) in the abdominal wall and PDAC. SFTs are rare, malignant mesenchymal tumors that can arise anywhere in the body and range from low to aggressive behavior [4]. SFTs account for fewer than 2% of soft tissue tumors, with signs and symptoms of SFT depending on the location of the tumor [5]. Treatment involves surgery, radiation therapy, chemotherapy, and targeted therapy [5]. The co-occurrence of PDAC and SFT guided us to investigate possible associated genetic mutations between both tumors.

## Case presentation

**Figure 1:**
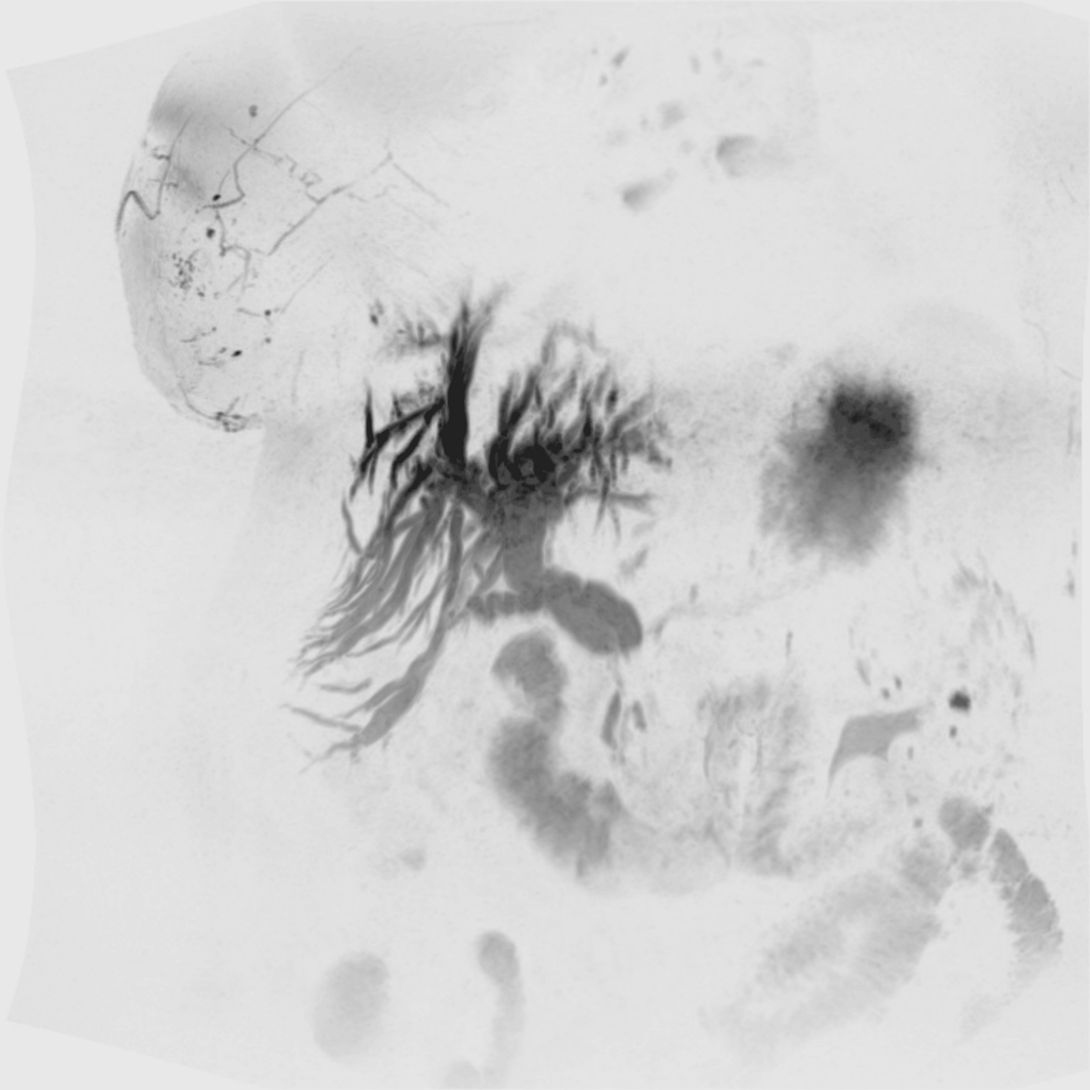
Magnetic resonance cholangiopancreatography showing intrahepatic biliary dilation, proximal common hepatic duct dilation, and a distal common bile duct stricture.

A 64-year-old woman with a history of chronic hepatitis B and prior cholecystectomy was brought to the emergency department, presenting with symptoms indicative of obstructive jaundice. These symptoms included severe abdominal pain, visible jaundice, and itching. Laboratory tests highlighted elevated levels of serum bilirubin, alkaline phosphatase, aspartate aminotransferase, and alanine aminotransferase. Investigations were initiated to determine the cause of the obstructive jaundice, effectively ruling out cholangitis. A magnetic resonance cholangiopancreatography scan subsequently identified a small cystic lesion in the pancreatic body and a narrowing in the common bile duct. An endoscopic retrograde cholangiopancreatography confirmed this narrowing, which was suspected to be cancerous (Figure 1).

**Figure 2:**
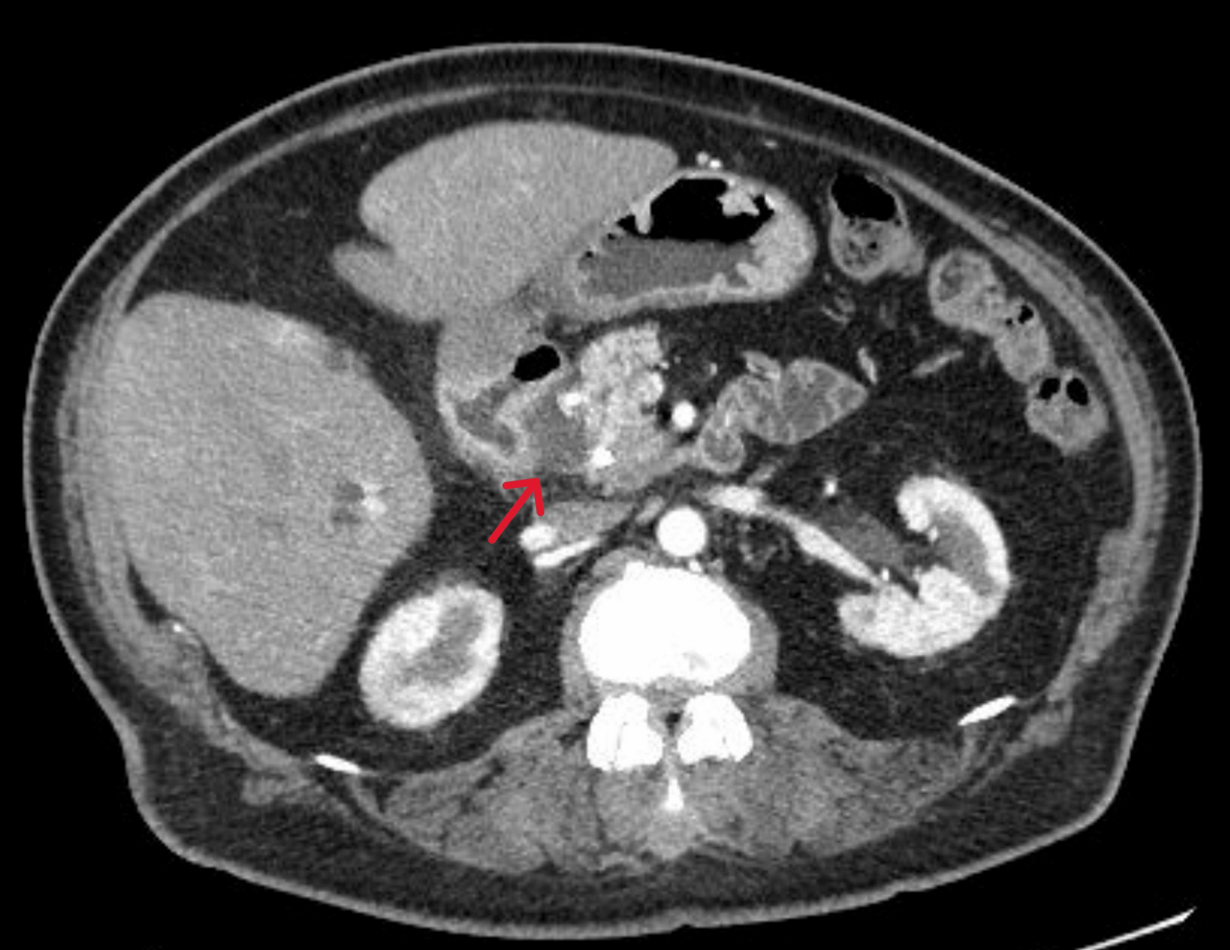
Abdomen CT with contrast showing the presence of a soft tissue mass (red arrow).

**Figure 3:**
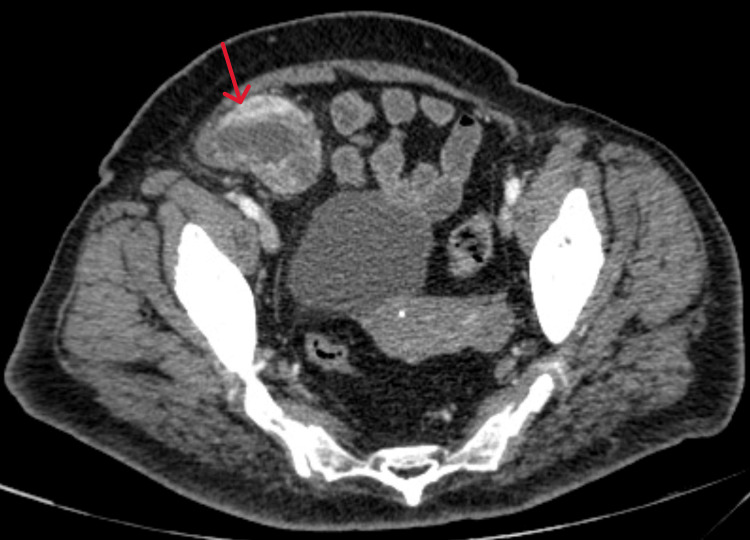
Pelvic CT with contrast showing the presence of a soft tissue mass (red arrow).

A CT pancreatic protocol was performed to reveal a mass in the head of the pancreas and a soft tissue mass with heterogeneous enhancement in the right hemipelvis measuring 8.6 × 7.8 × 5.9 cm. Abdomen and pelvic CT scans were also done showing the same results as the pancreatic protocol (Figures 2, 3).

**Figure 4:**
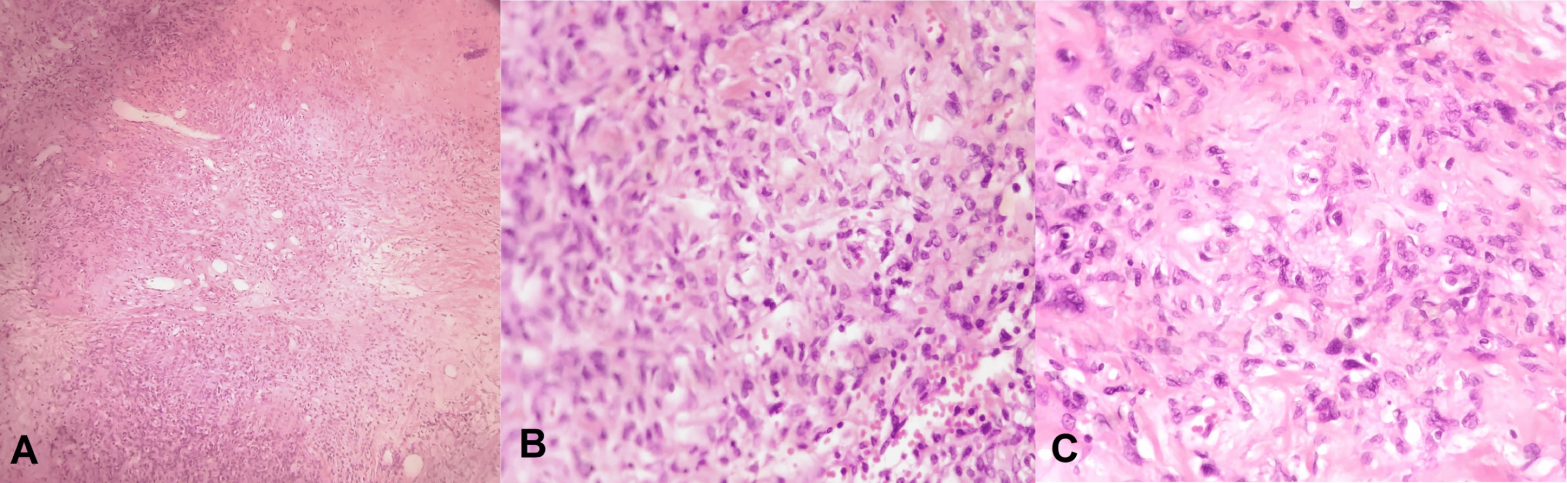
Histopathology of a solitary fibrous tumor. A-C: Well-demarcated, moderately cellular spindle cell tumor in a collagenous stroma with prominent dilated vessels (hemangiopericytoma-like vessels).

An excisional biopsy of the pelvic mass was undertaken for staging purposes. Gross examination of the biopsy sample presented a well-defined, white mass measuring 7 × 5 × 4.5 cm with a smooth cut surface. Histologically, the mass was found to consist of moderately dense spindle cells within a collagenous matrix, featuring dilated vessels reminiscent of hemangiopericytoma. Immunohistochemistry staining indicated positive CD34 expression, leading to a diagnosis of an SFT (Figure 4).

**Figure 5:**
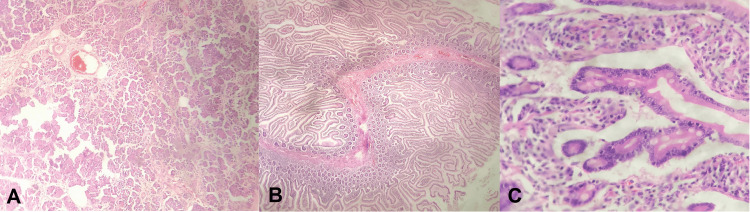
Histopathology of pancreatic ductal adenocarcinoma. A-C: Moderately differentiated pancreatic head adenocarcinoma measuring 2.5 × 2.5 cm, with focal tumor extension into the surrounding pancreatic tissue and surrounding fat.

Following confirmation of the benign nature of the pelvic mass three weeks later, the patient underwent a Whipple procedure to remove the PDAC, with histopathology confirming clear surgical margins (Figure 5).

Genetic testing was also performed, analyzing sequence and deletion/duplication in 10 genes associated with various genetic disorders, including *ATM*, *BRCA1*, *BRCA2*, *CDKN2A* (p14ARF), *CDKN2A* (p16INK4a), *MLH1*, *MSH2*, *MSH6*, *PALB2*, *PMS2*, and *STK11*. This analysis did not reveal any pathogenic variants.

Post-excision of both masses, the patient received adjunctive radiochemotherapy for pancreatic cancer. She made a good recovery from the surgery, with no signs of recurrence noted during the follow-up nearly a year later.

## Discussion

SFT and pancreatic cancer are distinct neoplasms that have never been reported concurrently in the same patient. This phenomenon has been scarcely reported in the literature, with only one documented case of cerebral SFT and pancreatic cancer co-occurrence in the same patient to date [6]. Zhou et al. reported the case of a 32-year-old woman who presented with epigastric pain and an abdominal mass, which upon imaging revealed a 130 mm pancreatic tail mass with distinct characteristics. Pathological examination later confirmed it as an anaplastic carcinoma with osteoclast-like giant cells. Notably, the patient had a history of recurrent SFTs [6]. Here, we present a case of synchronous benign SFT and PDAC. The rarity of such co-occurrence prompted an investigation into potential underlying genetic mutations linking these two distinct tumors. Despite limited data on concomitant SFT and pancreatic cancer, both lesions have been reported separately in the literature.

Overview of genetic mutations in pancreatic cancer

Pancreatic cancer is the third most common cause of cancer-related mortality in both genders, with mortality rates gradually increasing in men, but remaining relatively stable in women over the years [7]. Shifting our focus to the most prevalent genetic driver mutations that have been reported in pancreatic cancer, while most patients with pancreatic cancer have no single identifiable cause, somatic genetic mutations of pancreatic cancers are extensively studied. Notably, pancreatic cancer exhibits recurrent mutations in key genes such as *KRAS*, *TP53*, *CDKN2A*, *SMAD4*, *RNF43*, *ARID1A*, *TGFβR2*, *GNAS*, *RREB1*, and *PBRM1* [8]. Additionally, other mutations have been observed in known oncogenes, DNA damage repair genes, and chromatin modification genes at slightly higher false discovery rates [8]. Activating *KRAS* mutations are the defining genetic feature of PDAC progression and are present in approximately 92% of PDAC cases [9].

Numerous germline mutations associated with pancreatic cancer have also been identified. Approximately 20% of pancreatic cancer patients with a significant family history or personal history of malignancy have an identifiable causative germline mutation [10]. Moreover, germline mutations in pancreatic cancer susceptibility genes have been detected in patients without significant familial cancer history. In a study of 854 pancreatic cancer patients, 3.9% harbored pathogenic germline mutations, of which only 9% had a family history of pancreatic cancer [11]. Germline mutations in *BRCA1*, *BRCA2*, *ATM*, *PALB2*, *CDKN2A*, *STK11*, *TP53*, and mismatch repair genes (*MLH1*, *MSH2*, *MSH6*, *PMS2*, and *EPCAM*) are among the well-known inherited susceptibility genes [12]. However, genetic testing results for our patient revealed no pathogenic variants. The analysis included sequencing and deletion/duplication screening of 10 genes associated with various genetic disorder*s*:* ATM*,* BRCA1*,* BRCA2*,* CDKN2A *(*p14ARF*), *CDKN2A* (*p16INK4a*),* MLH1*,* MSH2*,* MSH6*,* PALB2*, *PMS2*, and* STK11*.

In the International Agency for Research on Cancer germline *TP53* database, pancreatic cancer occurred in 1.2% of the individuals with *TP53* germline mutation, with a median age of 53 years at diagnosis [13].

The genetic landscape of solitary fibrous tumor

SFTs are rare slow-growing neoplasms of mesenchymal origin that are initially identified in the pleura, mediastinum, and lung [5]. Occasionally, SFTs may occur in locations outside the thorax, such as the upper respiratory tract and retroperitoneum [14]. The discovery of the disease-defining *NAB2-STAT6* gene fusion resulting from an intrachromosomal inversion on chromosome 12q13.3 has provided valuable insights into unifying tumors with various histological variations, thereby improving diagnostic methods. Furthermore, the derived immunohistochemical detection of nuclear *STAT6* expression is highly valuable for distinguishing SFTs from other histologic mimics [15]. In a study by Hajdu et al., SFTs exhibited specific gene expression patterns compared to other sarcoma types. Upregulated tyrosine kinases, such as *FGFR1*, *ERBB2*, *DDR1*, and *INSR*, were observed in the SFT group. Several non-kinase genes, including *ADLH1A*, *IGF2*, *CHI3L1*, *GRIA2*, *FGF2*, and *ApoD*, were significantly upregulated in SFTs [16].

*TP53* is the most commonly mutated gene in human cancer [17]. Park et al. investigated metastatic tissue from SFTs and identified various molecular alterations, including mutations in *TP53* and *APAF1* genes. The study found that *TP53* immunopositivity and loss of *APAF1* immunoreactivity were significantly associated with malignant SFTs. Additionally, low *APAF1* expression was significantly associated with tumor malignancy and a higher rate of recurrence and metastasis [18]. The first case report providing evidence of the contributory role of *TP53* mutation in the development of dedifferentiated solitary fibrous tumor (dSFT), by describing a case of a unique and rare presentation of dSFT in the nasal cavity, was in 2011 [19].

By contrast, some germline mutations have been identified in different types of soft tissue sarcomas (STSs), as a large study was performed by the International Sarcoma Kindred Study, in which a kindred-oriented cohort of 1,192 sarcoma probands were interrogated for germline mutations in a panel of cancer-associated genes [20]. They reported that 55% of sarcoma cases harbored at least one pathogenic mutation [20]. Another study investigated an Asian cohort of sarcoma patients and identified germline mutations in cancer predisposition genes such as *RB1*, *DICER1*, *TP53*, *BRCA2*, and other DNA repair genes [21]. The study confirmed that a large number of reportedly sporadic sarcomas may harbor a germline component [21]. However, to date, there are no comprehensive reports of well-established germline mutations directly associated with SFT. To our knowledge, there is only one study that reported a case of SFT in a patient with germline mutation, synchronous with breast cancer [22]. This lack of documented germline mutations in SFT may imply their infrequency, but it does not rule out the possibility of their existence.

Furthermore, despite thorough sequencing studies, no significant point mutations, whether germline or somatic, were identified in frequently mutated cancer-associated genes such as *KRAS*, *BRAF*, and *PIK3CA*, concerning SFT [23,24].

Potential genetic links between solitary fibrous tumor and pancreatic cancer: germline *TP53* mutation discussion

A study found that among individuals with germline *TP53* mutations, sarcomas accounted for 25% of tumors [25]. Overall, 1.2% of the individuals with positive *TP53* mutations were also found to have pancreatic cancer [13]. One notable finding was the identification of a germline *TP53* mutation in a patient with SFT, which was also associated with Li-Fraumeni syndrome (LFS) [22]. Guha et al. presented a patient with a positive family history of cancer who was diagnosed with both malignant SFT and luminal B-like invasive breast cancer, which are both associated with LFS. The next-generation sequencing analysis revealed the presence of a rare genetic variant in the *TP53* gene, specifically at position c.1101-1G>A. This variant affects the acceptor splice site located in intron 10 of the *TP53* gene [22]. This observation may offer a potential link between the familial occurrence of pancreatic cancer in this syndrome and the development of SFT. Despite the absence of shared genetic mutations between SFT and pancreatic cancer in our findings, the identification of this unique case urges further exploration of the link between germline *TP53* mutations and the development of SFT.

The complexities of genetic associations between these different tumors indicate a need for larger studies and collaborative efforts that could provide a comprehensive understanding of the genetic basis of both tumors which could lead to more effective management approaches and treatment outcomes.

## Conclusions

To our knowledge, this is the first reported case of the synchronous presentation of an SFT and pancreatic cancer. However, the diagnosis was challenging as we initially suspected a metastatic tumor of pancreatic cancer, but eventually, we discovered it was a benign solitary tumor. Despite the negative result of the genetic study for this patient, further research may uncover a potential linkage between the two tumors.
